# Chicago Dental Society

**Published:** 1867-06

**Authors:** 


### CHICAGO DENTAL SOCIETY.

At the annual meeting of this Association, held on the evening of April 9th, the following officers were elected for the ensuing year:

*President.*—Dr. S. B. Noble.

1*st Vice-President.*—Dr. A. W. Freeman.

2*d Vice-President.*—Dr. J. H. Young.

*Secretary.*—Dr. A. E. Brown.

*Treasurer.*—Dr. Wm. Albaugh.

*Librarian.*—Dr. W. S. Stephens.

*Executive Committee.*—Dr. G. H. Cushing, Dr. J. W. Ellis, Dr. M. S. Dean.

